# Influence of Key Health Related Indicators on Adult Mortality: Result from UN Member Countries

**Published:** 2018-06

**Authors:** Chhabi Lal RANABHAT, Myung-Bae PARK, Chun-Bae KIM, Chang-Soo KIM, Hyoung-Sun JEONG, Sang Baek KOH, Sei-Jin CHANG

**Affiliations:** 1. Institute for Poverty Alleviation and International Development, Yonsei University, Wonju, Gangwon-do, Korea; 2. Health Science Foundations and Study Center, GPO–44600, Kathmandu, Nepal; 3. Manmohan Institute of Health Science, Kathmandu, Nepal; 4. Dept. of Gerontal Health and Welfare, Pai Chai University, College of Howard, Daejeon, Korea; 5. Dept. of Preventive Medicine, Wonju College of Medicine, Yonsei University, Wonju, Gangwon-do, Korea; 6. Dept. of Business Administration, College of Government and Business, Yonsei University, Wonju, Gangwon-do, Korea; 7. Dept. of Health Administration, Yonsei University, Wonju, Gangwon-do, Korea

**Keywords:** Adult mortality rate, Health indicators, Universal health coverage, Record linkage

## Abstract

**Background:**

Adult mortality is associated with different demographic and behavioral risk factors including approaches to health care financing. Adult mortality rate significantly reflects the effectiveness of public health-related program and intervention. The aim of this study was to find strength of association between key health’s related indicators and adult mortality rate.

**Methods:**

This cross-sectional study used 5 sets of data combined into one from different organizations of 193 countries using record linkage theory. Eleven key health-related indicators were taken as independent variables and adult mortality of male and female were dependent variables from 2010 to 2013. Average mortality for male and female was shown by means and standard deviations, raw association by Pearson correlation and strength of association by hierarchical linear regression.

**Results:**

The average adult mortality rate (AMR) of male was 0.209±0.106 and of female, 0.146 ±0.105 in years. In raw correlation, almost all health indicators were associated with AMR of male and female. In regression analysis, Universal Health Coverage (UHC) significantly reduced (male ∼0.43, female ∼0.30) adult mortality, in contrast, population growth significantly increased (male ∼ 0.37, female ∼0.43). Alcohol consumption per year increased AMR in male by 0.41 (*P*<0.01) and vaccination coverage (DPT 3) significantly reduced the AMR (0.26) in female.

**Conclusion:**

It is necessary to extend the UHC in remaining countries and still a need to control the population where there is high population growth. Effectively control of alcoholic drink in male and full coverage of vaccination in childhood mitigates adult mortality. The UHC is ambitious goal for SDG and special attention should be provided nationally and globally

## Introduction

Adult deaths threaten the livelihood of entire families (1) and seriously affect the children’s development (2,3) limit support on elderly (4,5) and economic activities of households (6). There is massive inequality in adult mortality. In some countries, the adult mortality is even higher after 2010 than that of 1990 and by sex, there is the highest women mortality in South Asia (7). Lesotho has the highest (0.50) and San Marino has the lowest (0.05) male adult mortality rate which is 10 times gap each other (8). Similarly, female adult mortality rate is 23 times higher in Lesotho (0.50) in comparison with Honduras (0.02) (9). South African experience shows that adults could be protected with healthy lifestyle and behavior, disease prevention approaches and adult friendly health policy (10). There are many factors associated adult mortality, which is different from neonatal, infant and maternal mortality. The risk factors; injuries, lifestyle, sexual behavior and other demographic factors were explored (11) but the situation of adult mortality with different varieties of health indicators have seldom been discussed.

The concept of adult mortality rate was developed in Europe to investigate the economic value of human in productive age (12). In comparison with all mortality, adult mortality is more important in term of national economy, sustainable development and family protection (13) and adult mortality is higher than other mortality due to more exposure with life-threatening events and injuries (14). Studies in adult mortality rate; (important health indicator) are not enough and the relation with related programs and indicators are extremely dearth. Health status, risk and outcome indicators are necessary to set the priority and long-term health planning (15) and WHO highlighted that health indicators have significant role in sustainable planning and development (16). A family health survey in India suggested that adult behavioral indicators (17) and sanitation indicators with infectious disease (18) are associated with adult mortality. To reduce adult death, it is necessary formulate special policy but before this, it is obligatory to find out the association between health-related indicator and adult mortality.

Global, regional and national adult mortality show that infectious diseases, major noncommunicable diseases like heart problems, cancer, injuries, poor immunity in childhood and adapted health care system have strong influences on adult mortality (19–21). Hygiene and sanitation, alcohol and tobacco use, child vaccination and health care expenditure explicitly determine the adult mortality. Socio-behavioral factors and biological factors (22,23) were risk factors for adult death. Education, which is the means for life, affects the mortality and morbidity of adults (24,25). The structural factors like social conflicts, economic disparities, and health financing police are strongly associated with adult mortality (26). Health expenditures are strongly associated with health outcomes and especially with reduction in child and adult mortality (27). The association between health indicators and quality of life was explored (28) however there was not a lot of research using that model.

The study and analysis of adult mortality tend to focus on risk factors and disease burden. In other words, research on adult mortality has been limited to individual factors but indifferent to program and indicators. There could be multiple associations between different health indicators like social determinants, human development and universal health coverage (UHC) (29) so that it is possible to access the proxy effect among them. This study will observe the association between demographics, diseases prevention, lifestyle and health financing indicators with adult mortality in a global context among UN country members. Probably, it is a first study to observe the vital health-related indicators for adult mortality and using data from diverse sources in different periods.

Therefore, the aim of this study was to find the strength of association between key health-related indicator and adult mortality from UN country members.

## Materials and Methods

### Study design

In this multi-country cross-sectional study variables from different organizations and timeline were used. The outcome variable, adult mortality rate was for the 2012–13 period and input variables prior to 2013.

### Data adoption model and sources

Data were created based on the Record Linkage theory applied by Dunn HL in 1946 (30). The objectives of this model were to preserve the records, verify them and create the new statistics, however, those data originated in different purpose. Furthermore, this model was advanced by Jutte DP 2011 as Administrative Record Linkage as a tool for public health research (31). We made the database from World Bank health data 2013 (32), WHO health report tobacco epidemic (33) and alcohol information (34) and “The political economy of UHC (35) (Table 1).

**Table 1:** Source of database

***S.N***	***Data sources***	***Reference***	
1	World Bank health data	World Bank: Data Catalogue	*Full complete data set*
2	Category of countries adopted and achieved universal health coverage	Stuckler D et al. The political economy of universal health coverage; 2010	
3	WHO health report tobacco epidemic 2013	World Health Report 2014	
4	WHO alcohol information report 2010	World Health Report 2014	

### Data management

We created new data by country for all 193 UN members. Data and outliers were cross-checked and missing data from some countries were excluded from the study. After verification and re-verification, the data were exported into SPSS -20 version (Chicago, IL, USA).

Full complete data set

### Data analysis model

1. The descriptive results were presented with means and standard deviations as global average.
2. In the second phase, Pearson correlation coefficient was computed.
3. In third step, multi-variant analysis was performed using hierarchal linear regression models

The data were interpreted by beta coefficient and *P*-value at the level of <0.05 and <0.001. The conclusion was drawn from the results hierarchical linear regression full model.

### Structure of variables

All of the above variables are numerical except universal health coverage. UHC is combined with 3 indicators; legal obligation of UHC, skill birth attendance rate and health insurance coverage. Therefore, we applied the UHC as yes =1 and no =0 category. After making consistent variables, we performed the linear regression models.

### Validity and reliability

We verified Word Bank, WHO and UNDP data used for this study. Likewise, the normality of data was checked by the observation of histogram and consistency of data was checked by Cronbach’s alpha for appropriate variables. Universal health coverage is very wide concept and needs to check multicollinearity. Multicollinearity was checked by variance inflation factors (VIF) with the standard of less than 3.

### Ethical aspects

The data is open access to everyone from above organizations and no need to take ethical approval.

## Results

### Descriptive statistics

Fifty-eight countries (30%) have achieved universal health care. Primary school enrollment and child vaccination (DPT-3) was almost achieved (>85%). Average population growth was less than 2%, more than 1/3^rd^ population did not achieve sanitation coverage and out-of-pocket payment for treatment was 61.1%. The average adult mortality rate of both sexes was 0.167±0.108 and male and female AMR was 0.209± 0.106 and 0.146± 0.105 (Table 2).

**Table 2:** Descriptive status of all health indicators

***Variables (in years)***	***No. of countries***	***Mean ± SD***
Net primary school enrollment (NPSE - 012) %	147	95.8±31.0
Economic Growth Rate (EGR 06 - 010) %	177	3.4±2.3
Population Growth Rate (PGR - 012) %	191	1.4±1.3
Child Vaccination Coverage (DPT 3 - 012) %	189	89.2±13.0
Sanitation Coverage (SAN COV – 012) %	193	61.2±27.3
Average adult consumption (ALCOHOL CON - 010) in liter/year	174	5.9±3.9
Prevalence of adult smoking - male (Prev. AS male - 012) %	134	32.3±12.6
Prevalence of adult smoking - female (Prev. AS female - 012) %	134	11.2±10.4
Out of Pocket Payment (OPP - 012) %	185	61.1±39.5
Total Health Expenditure (THE - 012) %	184	6.7±2.9
Government Health Expenditure per year (GHE - 012) %	186	11.6±4.9
Adult mortality both sex (012)/thousand/year	181	167.1±108.8
Adult mortality rate-male (012) / thousand/year	181	209.9±106.1
Adult mortality rate-female (012) / thousand/year	181	146.3±105.1
Achievement of Universal Health Care (UHC) –Yes (N_1_ - 193)	58	30%

Cronbach's alpha 0.712, Variance inflation factors (VIF) with UHC and other factors <1.5

Table 3 shows the correlation status of all variables. Adult male mortality is positively associated population growth rate, alcohol consumption per year, prevalence adult smoking, out of pocket payment and government health expenditure (*P*<0.01). Likewise, it is negatively associated with economic growth rate, DPT 3 and sanitation coverage and universal health coverage. Similar trend can be observed in female adult mortality status. Female adult mortality had a strong positive association with population growth and strong negative association with DPT 3, sanitation and universal health coverage.

**Table 3:** Situation of raw correlation among all variables using Pearson’s correlation (*P-value<0.05, **P<0.01)

***S. N***	***Variables***	***1***	***2***	***3***	***4***	***5***	***6***	***7***	***8***	***9***	***10***	***11***	***12***	***13***	***14***
1	NPSE (012)	1													
2	EGR (06 - 010)	.213^*^	1												
3	PGR (012)	−.109	.349^**^	1											
4	DPT3 (012)	.046	−.146	−.335^**^	1										
5	SAN COV (012)	.065	.021	.150	.294^**^	1									
6	ALCOHOL CON	−.240^**^	−.282^**^	.194	.192	−.029	1								
7	Prev. AS – male (012)	−.048	.023	.523^**^.	.131	.152^*^	.533^**^	1							
8	Prev. AS - female (012)	−.078	−.411^**^	399^**^	.102	.151	0.24	.318^**^	1						
9	UHC	.003	.241^**^	−.272^**^	.292^**^	.147^*^	−.137	−.107	−.505^**^	1					
10	OPP (012)	−.074	−.204^**^	.358^**^	.114	.053	.381^**^	.037	.502^**^	−.202^**^	1				
11	THE (012)	.028	−.051	−.051	.030	.011	.071	.023	.082	.098	.116	1			
12	GHE (012)	.003	−.128	−.273^**^	.205^**^	.101	−.245^**^	−.079	−.359^*^	.240^**^	.598^**^	−.101	1		
13	AMR- male (012)	−.031	−.250^*^	.533^**^	−.480^**^	−.492^**^	.575^**^	.407^**^	.180	−.597^**^	.351^**^	.085	.336^**^	1	
14	AMR- female (012)	−.010	−.231^**^	.498^**^	−.514^**^	−.467^**^	.168	.258^*^	.304^**^	−.554^*^	.294*	.055	.271^*^	.608^**^	1

### Adult mortality by hierarchical regression model

Table 4 shows the association between all predicted indicators and male adult mortality.

**Table 4:** Linear regression among demographic, disease prevention, lifestyle, health financing indicators and male adult mortality rate

***Predictors***	***Adult mortality rate male (012)***							
	***Model 1***		***Model 2***		***Model 3***		***Model 4 (full)***	
	**St beta Coeff.**	**Sig.**	**St Beta Coeff.**	**Sig.**	**St Beta Coeff.**	**Sig.**	**St Beta Coeff.**	**Sig.**
**Eco-demographic indicators**								
NPSE (012)	−0.043	0.597	−0.010	0.894	0.068	0.479	0.100	0.265
EGR (06- 010)	0.082	0.396	0.111	0.236	0.141	0.209	0.064	0.561
PGR (012)	0.499	<0.001	0.380	<0.001	0.475	0.004	0.379	0.013
**Disease prevention indicators**								
DPT3 (012)			−0.265	0.003	−0.199	0.069	−0.154	0.120
SAN COV (012)			−0.131	0.115	−0.188	0.066	−0.096	0.298
**Lifestyle indicators**								
Adult smoking male					0.099	0.368	0.079	0.429
ALCOHOL CON					0.279	0.034	0.419	0.002
**Health financing indicators**								
UHC (Yes/No)							−0.433	<0.001
OPP (012)							−0.108	0.358
THE (012)							−0.039	0.648
GHE (012)							−0.037	0.723
R^2^	0.301		0.323		0.346		0.517	
Adj R^2^	0.274		0.275		0.283		0.448	
F value	10.355		9.846		5.776		6.912	
*P*-value	<0.001		<0.001		<0.001		<0.001	

Note: The details of variables have been presented in methodology and Table 2.

For the full model (model 4), AMR would significantly increase by 0.379 and 0.419 with population growth rate (*P*<0.05) and alcohol consumption per year. In contrast, AMR would significantly decrease (*P* <0.01, β ∼ 0.433) with universal health coverage.

In regression, the determinants health indicators of female adult mortality were little different. Lifestyle indicators were not associated with the full model but in model 3, female smoking prevalence was positively associated with mortality (*P*<0.05). In model 4 (full), female adult mortality was significantly increased (*P*<0.01, β ∼ 0.437) with population growth rate and significantly decreased with DPT 3 coverage (β ∼ 0.262) and universal health coverage (β ∼ 0.301) (Table 5).

**Table 5:** Linear regression among demographic, disease prevention, lifestyle, health financing indicators and female adult mortality rate

***Predictors***	***Adult mortality rate female (012)***							
	***Model 1***		***Model 2***		***Model 3***		***Model 4 (full)***	
	**St Beta Coeff.**	**Sig.**	**St Beta Coeff.**	**Sig.**	**St Beta Coeff.**	**Sig.**	**St Beta Coeff.**	**Sig.**
**Eco-demographic indicators**								
NPSE (012)	−0.076	0.314	−0.046	0.521	0.094	0.260	0.078	0.352
EGR (06 - 010)	0.018	0.839	0.035	0.680	−0.064.	0.503	−0.086	0.398
PGR (012)	0.586	0.000	0.463	0.000	0.490	0.000	0.437	0.001
**Disease prevention indicators**								
DPT3 (012)			−0.223	0.006	−0.273	0.005	−0.262	0.008
SAN COV (012)			−0.188	0.014	−0.134	0.129	−0.106	0.224
**Lifestyle indicators**								
Adult smoking female					0.242	0.037	0.171	0.166
ALCOHOL CON					0.198	0.069	0.188	0.126
**Health financing indicators**								
UHC (Yes/No)							−0.301	0.003
OPP (012)							0.127	0.270
THE (012)							−0.024	0.758
GHE (012)							−0.045	0.634
R^2^	0.331		0.429		0.521		0.576	
Adj R^2^	0.334		0.404		0.477		0.511	
F value	19.721		17.525		11.965		8.848	
P value	<0.001		<0.001		<0.001		<0.001	

Note: The details of variables have been presented in methodology and Table 2.

## Discussion

Adult mortality is directly related to disease or condition of the individual but adult mortality rate is a key health indicator and associated with other related indexes. The average male adult mortality was 1.43 times higher than females (Table 2). In the US (36) and Japan (37), due to several risk factors for diseases and injury, adult mortality was higher in men. In raw correlation, adult mortality of both sexes was correlated with almost independent variables however, the result was interpreted by linear regression full model (model 4). Population growth rate, alcohol consumption, universal health coverage and vaccine coverage (DPT 3) childhood are strongly associated (*P*<0.05) with adult mortality rate among 11 major indicators in linear regression (full model). In other words, this study is more useful to observe related program impact on adult mortality.

There is a linear positive association between population growth rate and adult mortality for men and women. The adult mortality of men and women would increase by 0.379 and 0.437 with population growth rate. This estimation is similar to the demographic transition formula (38) and population estimation (39). The adult mortality is high in South Asia, Africa and Middle East Asia where population growth is also high (40). Similarly, there is linear negative association between universal health care and adult mortality status (26). The adult mortality would decrease by 0.433 and 0.301 respectively for men and women with universal health coverage as like above study. UHC overall improves the population health by equity and access of preventive, promotive, curative and palliative health care without financial constraints (41) and health program like vaccination, sanitation, maternal and child health, nutrition, health education etc. are assured so that the adults could be strong from childhood. As a health care service modality, UHC with universal cooperative health insurance would reduce adult death (42).

Among men, the adult mortality is significantly higher (*P*=0.002, β∼0.41) with alcohol consumption. There is no established causal relationship of alcohol consumption with fatal diseases but male drinking behavior would be responsible. Usually, male adult mortality increases with heavy and uncontrolled drinking behavior (43,44) and this behavior has multiple impacts; increasing violence and murder, worsening of infectious diseases like tuberculosis and hepatitis, limiting life expectancy with liver and heart diseases, increasing mental diseases and suicide and increasing unintentional injuries due to drunk driving (45). Moreover, unprotected sex behavior is common after alcohol consumption and the risk for sexually transmitted diseases particularly HIV is increased (46). In South Africa, alcohol users were more vulnerable to risky sexual intercourse (47) and the prevalence of adult mortality was high in Africa due to this circumstance.

The determinant indicators for male and female adult mortality are different. Child vaccination DPT -3 would reduce female adult mortality by 0.262 (*P*<0.01). The girl’s death rate was 2 times higher for those not received the DPT vaccine in comparison with those who received the vaccine (48) which is similar to our findings. However, DPT vaccine was associated with higher mortality for girls (49) and obviously could not protect through the adult stage. The coverage of this study was wide and included all UN country members and 5 different sources of data. Major determinant indicators of adult mortality rate were examined however, there were not equal samples for all variables examined and those countries numbers may not enough from statistical perspective.

## Conclusion

Population growth rate and alcohol consumption amount would increase the adult mortality rate but it can be reduced by universal health coverage. The UHC is ambitious goal for SDG and special attention should be provided nationally and globally.

### Ethical considerations

Ethical issues (Including plagiarism, informed consent, misconduct, data fabrication and/or falsification, double publication and/or submission, redundancy, etc.) have been completely observed by the authors.
